# Commentary: Review of Mandatory Maintenance of Certification in the USA

**DOI:** 10.1177/01632787241311360

**Published:** 2025-01-03

**Authors:** Katya Peri, Mark J. Eisenberg

**Affiliations:** 1 5621 Jewish General Hospital, Canada; 2 5620 McGill University, Canada

**Keywords:** commentary, career development, medical education, continuing education, recertification

## Abstract

The goal of maintenance of certification (MOC) activities is to ensure physicians are up to date on current practices and demonstrate the knowledge and skills required to provide patients with optimal care. The program’s aim is to promote professional development, lifelong learning and quality assurance for the public and medical community. However, physicians are not happy with the current structure of the program, claiming it to be time-consuming, expensive and ineffective for their practice. The lack of concrete evidence confirming the efficacy of MOC in improving knowledge and clinical outcomes causes many to question how this system can be improved to better serve practitioners and the public. In this commentary, we provide an overview of the current MOC situation for U.S. specialists and highlight the importance of increasing research to inform evidence-based changes that can be applied to clinical situations.

## A US Historical Perspective

For physicians in the U.S., the American Board of Internal Medicine (ABIM) is the main certifying board from which specialists are certified. Currently certifying over 25% of physicians, its mission is to, “enhance the quality of health care by certifying physicians who demonstrate the knowledge, skills and attitudes essential for excellent patient care.” (Baron et al., 2015). This organization actively embraces public reporting and public accountability and seeks to create standards that render physicians accountable for their quality of care. To better serve the public and take accountability for their safety, board certification and recertification programs were introduced. These programs give physicians the opportunity to demonstrate their scholarity, leadership and dedication to the field and to their patients’ welfare. Previously, the ABIM offered a lifelong certification, however in 1969, they transitioned to a continuous learning approach and offered voluntary recertification (Meskauskas & Webster, 1975). During this time, less than 10% of eligible physicians opted to recertify themselves, leading them to enforce a ten- year recertification and continuing education program on physicians licensed after 1990 (Wasserman et al., 2000). In 2014, ABIM further transformed the program by imposing annual fees, continuous certification exams and a variety of educational modules.

**Figure 1.**
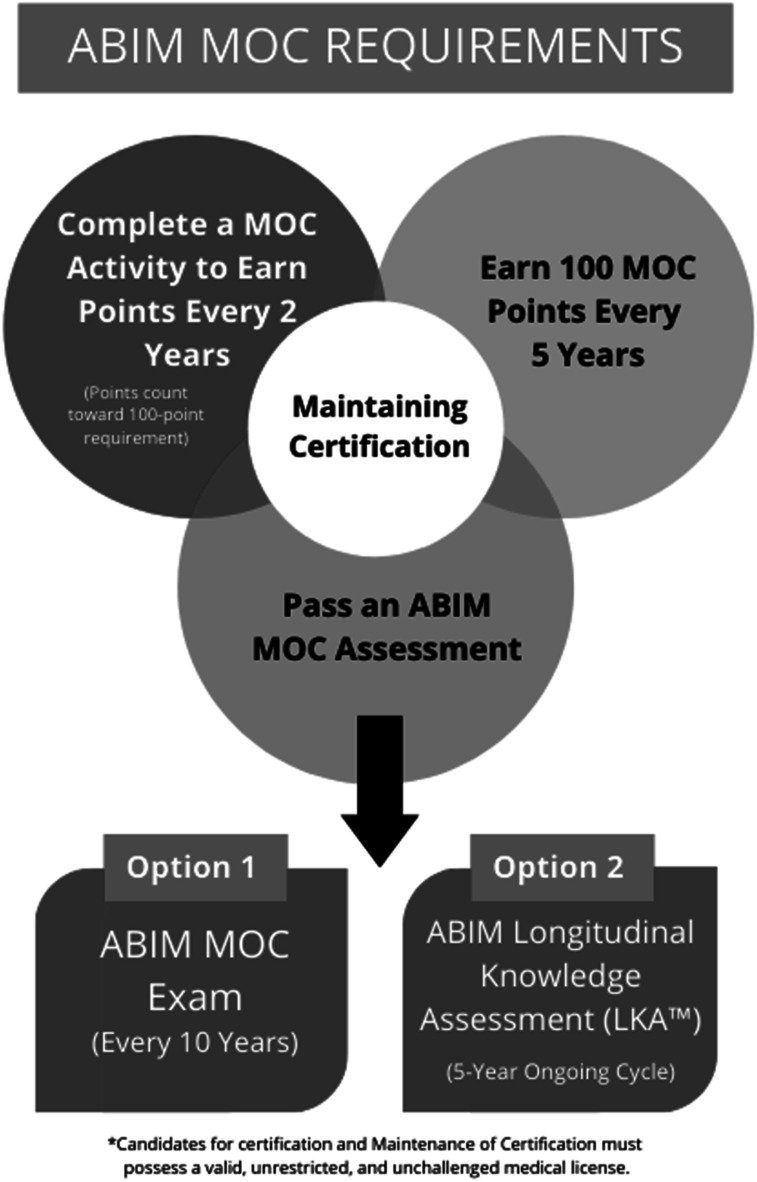
Flowchart Depicting Yearly Requirements and Options Available to Physicians to Pass Their MOC Requirements. Reproduced With Permission From the American Board of Internal Medicine

**Figure 2.**
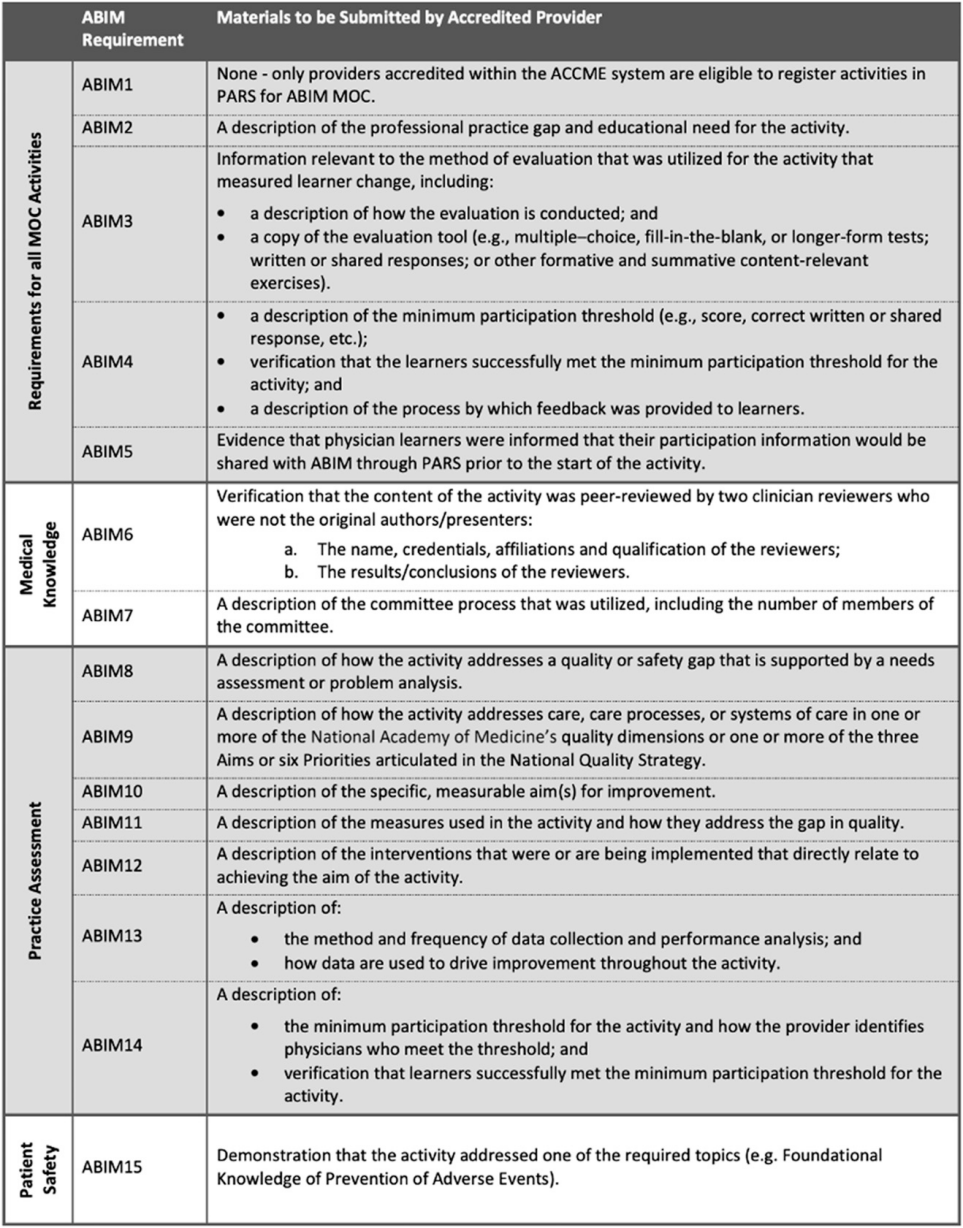
Required Materials and Requirements That Must be Met to Maintain Internal Medicine Certification. Reproduced With Permission From the American Board of Internal Medicine

**Figure 3.**
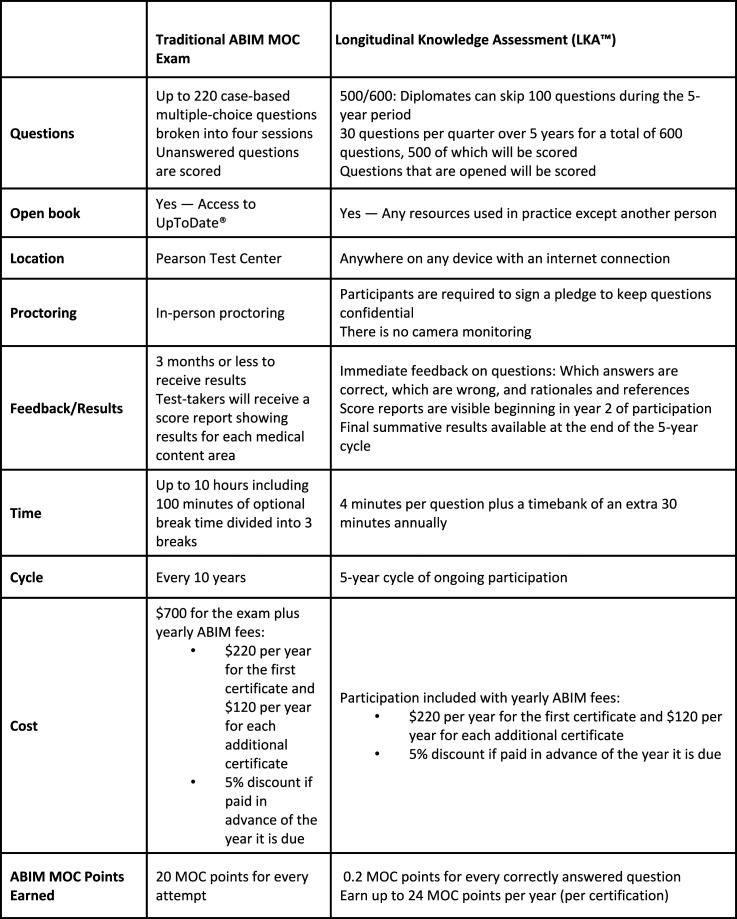
Comparison of Traditional ABIM MOC Cumulative Exam With the Newly Inaugurated Longitudinal Knowledge Assessment. Reproduced With Permission From the American Board of Internal Medicine

Today, specialists must partake in continuous professional development to maintain their certification and status. Previous studies have shown that physicians lack the ability to self-assess and are overconfident in their ability to diagnose disease (Davis et al., 2006; Eva & Regehr, 2008). Furthermore, formal examination guarantees the maintenance of clinical knowledge and the acquisition of skills regarding novel therapeutic techniques. In addition to recertification exams, the MOC process involves earning points by completing activities including courses, seminars, conferences, manuscript reviews, and teaching (Medicine, 2024). A physician must earn 100 points a year for a total of five years (500 points total) (Figure 1). The points are divided into specific categories: medical knowledge, patient safety and practical assessment with specific ABIM requirements numbered from 1–15 (Figure 2). All activities eligible for MOC accreditation must meet ABIM1-5 requirements while each of the three categories have specific requirements that must be additionally met. Furthermore, at least 20 of the 100 points earned every five years must be in the medical knowledge category. Two options exist for the assessment of clinical knowledge: the recertification exam (must be passed every ten years) and the longitudinal knowledge assessment (five-year ongoing cycle) (Medicine, 2024) (Figure 3). Failure to do so results in an exam rewrite and potential loss of certification should licensing criteria are not met. In addition to ABIM criteria, different states might have additional requirements physicians have to abide by to keep their certification, including limitations on license period, number of credit hours required for recertification and types of MOC required. However, not all physicians agree with the current structure of MOC and arguments for and against this practice emerge in the medical community.

## The Efficacy of MOC: A Subject for Debate

Over the past several years, the MOC process has been hotly debated among physicians and board members, who question the value of the program. In 2014, the ABIM reformed its MOC program to include annual fees, continuous education, and frequent assessments. Additional requirements are also necessary for physicians in certain specialties such as interventional cardiologists, and hospitalists such as procedure requirements. Physicians protested against the new requirements and petitions gathering more than 22,000 signatures were circulated amongst cardiologists (Teirstein & Topol, 2015). Their arguments involved the futility and time-consuming nature of the new guidelines as well as the limited evidence regarding the positive effects of this program. Disagreement surrounding this reform has led many to question if continuing medical education (CME) should be mandatory or voluntary.

Arguments in favour of mandatory continuous recertification focus on maintaining quality and competence in healthcare professionals. Questions of patient safety and physician knowledge brought to light by the Bristol Inquiry and the To Err is Human Report solidified the need for MOC to assure safe clinical practice (Dyer, 2001; Institute of Medicine Committee on Quality of Health Care in, 2000). It is well known that increased knowledge is directly associated with better clinical outcomes (Marinopoulos & Baumann, 2009). Interestingly, as the number of years since initial certification increases, the quality of psychomotor skills and clinical outcomes decreases (Ramsey et al., 1991). Previous studies have shown that recertified cardiologists have a 19% lower mortality rate in their practice compared to those who are not (Norcini et al., 2002). Furthermore, physicians undergoing MOC demonstrate improvement in knowledge, practical skills and long-term maintenance of information (Leopold et al., 2005; Marinopoulos et al., 2007). One systematic review evaluating the effect of MOC on clinical outcomes revealed that patients of 39% of physicians in the study experienced improved clinical outcomes for more than 30 days (Leopold et al., 2005; Marinopoulos et al., 2007). A systematic review including 39 studies examining the associations between MOC and physician’s knowledge and practice determined that MOC appears to help increase knowledge and improve behaviour (Price et al., 2018). Therefore, ongoing practice support can promote sustainable improvements in practice and patient outcomes. Furthermore, CME activities have been shown to help senior clinicians mitigate the age-related decline in diagnostic performance (Butterworth & Reppert, 1960). This decline can be in part due to automaticity, which prevents experienced clinicians from exploring new ways of treating patients due to routine (Cordovani et al., 2020). This can be a hindrance when incorporating new technology in practice. Furthermore, over time, a specialist’s practice can narrow down significantly. MOC ensures reinforcement of core medical competencies that may be lost over time.

In contrast, the efficacy and validity of CME and MOC have been questioned as the program continues to change and expand. Current CME systems operate on a credit-hour system where 1 hour of education is equal to one credit. However, some practitioners argue that the quantity of hours does not necessitate improvement in the quality of clinical performance or knowledge acquisition (Stodel et al., 2015). Despite evidence in favour of the MOC’s use in maintaining skill, there is also a significant amount of research proving the inefficacy and futility of this program. A meta-analysis reveals that despite 16 out of 33 studies showing a positive correlation between MOC and improved outcomes, 14 showed no association and three showed a negative association (Sharp et al., 2002). Furthermore, the quality of these studies was inadequate to draw concrete conclusions. This research promotes broad skepticism about the usefulness of MOC. Of note, there is no tangible evidence demonstrating that physicians who were “grandfathered” out of the MOC program due to certification before 1990 provide lower quality care than those who participate in MOC. The number of ambulatory care-sensitive hospitalizations, commonly used to measure preventable hospitalizations, did not decrease with providers taking MOC (Hayes et al., 2014). Furthermore, a study conducted in Veteran Affairs Medical Centers did not discern a difference in care between time limited and unlimited ABIM certification. In addition, physicians’ debate that enforcing MOC violates the autonomy of the profession, causing a loss of respect (Emanuel & Pearson, 2012; Mechanic, 2003; Reinertsen, 2003). Another criticism of the recertification exam is that it reinforces “binge studying”, or intense knowledge acquisition over a short period of time that does not translate to long-term maintenance or improved clinical skills (Valentine et al., 2019). This directly goes against the MOC’s role of promoting lifelong learning, quality assessment and improved care. In addition to this transgression, recertification exams are required to be completed without the use of outside sources (Iglehart & Baron, 2012). This goes against the pervasive idea in medicine that doctors should base their decisions with the help of the best sources and information available to them to provide the best care. Commercial bias and financial conflict are also concerns. Many physicians have close ties with certain pharmaceutical companies, which could cause conflicts of interest in diagnosis or treatment. Additionally, health insurance policies, hospital regulations, complex care coordination and lack of transparency among care teams are also barriers to quality patient care. Furthermore, the financial burden of MOC is significant, with most physicians paying 2200 – 3400$ a year to maintain their credentials26. For specialists, the average cost is approximately 23,000$ over 10 years and for subspecialists this rises to 40,000$. Physicians also complain that the process is not only expensive but very time-consuming, further adding to their reluctance to engage in MOC.

## The Future of MOC

The MOC program has been constantly changing since its inception and still requires quite a bit of reformation. Due to pressure from physicians and external stakeholders, the American Board of Medical Sciences has begun prioritizing evidence-based reformation and the integration of MOC into practical environments (Iglehart & Baron, 2012). This is essential to the improvement of the program, since current research on the effectiveness of MOC is limited and results are not clear. More research focusing on the effect of the program on patient health outcomes and the types of activities that are the most beneficial to physicians is needed to inform future changes. Members have stated that they are not against the idea of MOC, but rather the way it is employed. Passive learning methods, such as lectures, meetings, and grand rounds are time-consuming and do not encourage doctors to reflect on their own practice. The present structure is better equipped to grade physicians through summative assessment of previously acquired knowledge. But to operate optimally, CME should emphasize continuous education, knowledge interpretation, self-assessment, and practical learning, all competencies that are essential to the betterment of practicing physicians. New modules should focus on practical learning and self-assessment to help physicians identify and close knowledge gaps. Cooperation between candidates and board members is necessary to create a valuable recertification program relevant to everyday clinical practice. Coupled with constant research and evaluation on the MOC program, this new philosophy should create a program that benefits all involved.
